# Blockchain vehicles for efficient Medical Record management

**DOI:** 10.1038/s41746-019-0211-0

**Published:** 2020-01-06

**Authors:** Anuraag A. Vazirani, Odhran O’Donoghue, David Brindley, Edward Meinert

**Affiliations:** 1 Medical Sciences Division, University of Oxford, Oxford, UK; 2 Department of Paediatrics, University of Oxford, Oxford, OX3 9DU UK; 3 Department of Primary Care and Public Health, Imperial College London, London, W6 8RP UK

**Keywords:** Health care, Information technology

## Abstract

The lack of interoperability in Britain’s medical records systems precludes the realisation of benefits generated by increased spending elsewhere in healthcare. Growing concerns regarding the security of online medical data following breaches, and regarding regulations governing data ownership, mandate strict parameters in the development of efficient methods to administrate medical records. Furthermore, consideration must be placed on the rise of connected devices, which vastly increase the amount of data that can be collected in order to improve a patient’s long-term health outcomes. Increasing numbers of healthcare systems are developing Blockchain-based systems to manage medical data. A Blockchain is a decentralised, continuously growing online ledger of records, validated by members of the network. Traditionally used to manage cryptocurrency records, distributed ledger technology can be applied to various aspects of healthcare. In this manuscript, we focus on how Electronic Medical Records in particular can be managed by Blockchain, and how the introduction of this novel technology can create a more efficient and interoperable infrastructure to manage records that leads to improved healthcare outcomes, while maintaining patient data ownership and without compromising privacy or security of sensitive data.

## Background

The attempted reforms to Britain’s medical record systems in recent years have left an incompletely digitised complex: paper records remain ubiquitous at the secondary level, in tandem with several disconnected local Trust-specific electronic systems. Despite significant advances in the use of technology in clinical medicine and the large sums of money recently diverted into the National Health Service^1,2^, administrative systems in healthcare remain in want of interoperability.

This lack of interoperability can lead not only to clinical errors but administrative ones, such as the National Health Service’s (NHS’s) recent failure to invite 50,000 women for a cervical screening test^3^. Furthermore, patients must recount their history multiple times, a process found to be inefficient as well as tiresome, and which can lead to confusion as well as clinical errors because of incomplete information^4^.

More importantly, the lack of structure can lead to avoidable situations in which timely information is unavailable, that have clinical repercussions. Increasing numbers of systems use Blockchain in different ways to solve the problem of interoperability, by empowering doctors with more comprehensive patient data, acquired from connected but independently managed systems.

## Blockchain

A Blockchain is a decentralised database, the data within which is validated by members of the network^5^. It has traditionally been used to manage cryptocurrency transaction records, but can also be applied to various aspects of healthcare such as administration of prescriptions (and associated fraud prevention), insurance coverage, and electronic medical record management^6^. Instead of trusted third-party signatories (such as VISA in a financial context), Blockchain uses cryptographic proof to validate records. A network of users, collectively adhering to a set of pre-agreed rules, carries out this cryptographic validation. This introduces integrity, ensuring only one single ‘correct’ version of events is stored in the database, which cannot be changed subsequently without the agreement of a majority of nodes. This method works by locking each set of records in the database (termed a ‘block’) to the previous block with the use of a hash, such that a change in one block would modify the hashes of all subsequent blocks.

**Fig. 1:**
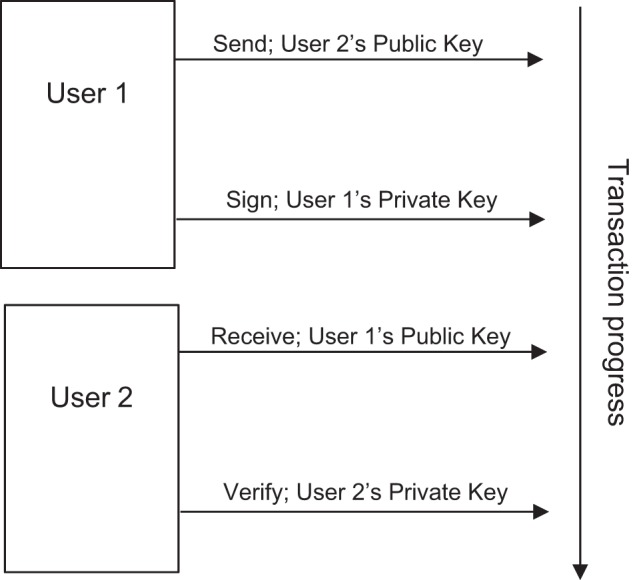
Progress of a Blockchain transaction. Public-key cryptography creates a public and a private key for each user, using a one-way hash function to create the public from the private key. The public keys are used by the sender and receiver of a transaction to identify each other. Private keys remain undisclosed, and are used by the sender and receiver to sign and verify transactions, respectively. Here, User 1 sends a transaction to User 2, using User 2’s Public key. User 2 receives the transaction, identified as having been sent by User 1’s public key.

As well as being at risk of integrity flaws, current healthcare management systems are vulnerable to cyber attacks, such as the WannaCry attack in 2017^7^ that affected computers in 80 of the 236 NHS trusts^8^, along with more than 250,000 computers in 150 countries. More recently, an attack on SingHealth, Singapore’s main healthcare group, compromised the data of 1.5 million Singaporean citizens^9^. In order to ensure that healthcare systems do not remain an accessible target for hackers, sufficient precautions must be put in place to protect patients’ sensitive data. Blockchain uses public-key cryptography to secure data: a public and a private key are generated for each user using a one-way encryption function (hash). These may be used by both parties in a transaction: the sender signs, and the receiver verifies, using their own private key, and public keys are used to send transactions to a recipient (Fig. 1). This allows the recipient to verify the validity of the chain of information. In addition, only the recipient can see the information sent, eliminating any possibility of hacking.

The system can also allow components of arbitrary logic to be added in order to process, validate, and sanction access to the data secured within, simplifying consent processes for patients and doctors. This is known as a smart contract, and functions as a string of computer code that executes whenever these certain predetermined conditions are met^10^, ensuring both the security of the system and authorised access. It is this ability to create smart contracts that makes Blockchain suitable for healthcare, a field in which strict regulations govern how sensitive data may be used^11,12^, an increasingly important factor following the recent introduction of the General Data Protection Regulation.

Another major concern relating to healthcare records is the cost associated with transferring records between locations^13^, and in particular between Trusts. Sending data via email is considered a security risk^14,15^, while there is clear inefficiency inherent in transcribing a digital asset onto optical media, which is commonly only read once at the receiving site^16^. Furthermore, repeated imaging studies carried out because of unavailability of prior results can be dangerous in the context of delayed treatment as well as financially costly. As a decentralised database, Blockchain is fundamentally interoperable, and authorised sharing of data comes at no extra cost.

## Implementation

Blockchain would most effectively integrate as a mode of managing access to sensitive health data, although in practise this could take many forms. In principle, by storing an index of health records and related metadata linking to the sensitive data (stored elsewhere on a secure cloud), the system would introduce a layer of interoperability to the currently disjointed set of systems^17,18^. This type of framework is exemplified by MedRec^19^, a system employed in Boston, which not only allows data to be accessed with consent by a patient’s multiple healthcare providers, but also accommodates access for epidemiological researchers.

**Fig. 2:**
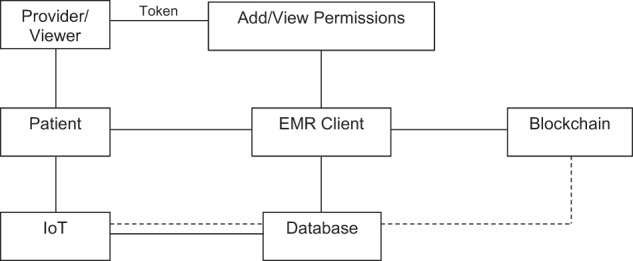
Interactions in the Blockchain-based healthcare system. Patients have full access to the data via the EMR Client. Data may be added or viewed by doctors and other providers, who require permissions to do so, and added by devices including wearables, which form a part of the Internet of Things (IoT). All interactions are stored on the Blockchain.

In this framework, a cloud-based medical record is associated with viewing permissions and data retrieval instructions, thus using the Blockchain to record patient–provider interactions via smart contracts. Once a doctor creates a record, it is verified, and its viewing permissions are authorised by the patient and stored in a smart contract. The record can never be modified without the agreement of a majority of nodes (inconvenient and probabilistically unlikely). Temporary access can be controlled by the use of temporary keys (‘tokens’), created by users and passed onto those such as healthcare providers and insurance companies (Fig. 2). The token is independent of the data, containing only authorisation commands, and is validated (by recording it on the chain) before the required reports are dispatched^20,21^.

## Integration

Twenty-first century healthcare data extends beyond the standard formats of written reports, DICOM (Digital Imaging and Communications in Medicine) standard images and basic lab results: wearable technology such as bracelets and watches, which can collect more regular information points than manual technology, are used increasingly. These enhance data collection either by increasing the frequency of data points or by delivering data in a more user-friendly format. A more recent example takes the form of Apple’s ‘iSheet’^22,23^, a patent for a bedding device, which will take continuous measurements of vital signs, as well as sleep pattern information. Should the extensive data from these new contributors to the Internet of Things (the growing network of connected, data-exchanging devices) be harnessed effectively, doctors may be able to provide more personalised care based on the individual’s detailed personal data. Data could even be analysed by artificial intelligence systems, in order to find as yet undetermined paradigms in personal health.

## Discussion

After multiple attempts and delays at becoming fully digital^24^, the NHS has now released a ten-year plan^1^, in which it introduces the basis for a new twenty-first century service model. This includes digitally equipping all primary and outpatient care, and empowering patients to access, manage, and contribute to digital information and services, which includes incorporating data added by patients themselves. In addition, they pave the way to improve quality of life for those with long-term conditions by the use of connected and interoperable devices.

Achieving interoperability, however, depends on patients taking control of their data and deciding on how it will be used. Data ownership would need to be shifted from the government to patients, and while this would require extensive reengineering of legacy systems, it would hopefully incentivise patients to become active agents in their own care. By contributing data to the system, they would be able to get the best possible treatment^25,26^, exemplifying the notion of patient-centred care. The re-engineering of systems would need to keep in mind legal restrictions, such as the recently introduced General Data Protection Regulation. Under this law, patients may request for their data to be erased^27^. However, with a Blockchain, a record of the data’s previous existence would always be maintained on the chain.

In addition to abiding by legal restrictions on data use, Blockchain would need to guard against intruders. Not only do data breaches cause damage by the loss of data to hackers, but they also have a negative impact on the public perception of the healthcare field, and threaten to hinder future research through more stringent regulatory restrictions^28^. While a Blockchain is more secure than older methods^29^, most are still susceptible to a ‘51% attack’, in which a majority of mining nodes collude to rewrite the chain structure^30^. Users must trust that at least 50% of mining nodes would not want to violate the immutability of the Blockchain. The use of a ‘permissioned’ (as opposed to permissionless) Blockchain, however, can allow a healthcare system to rule out any possibility of this style of attack. This method limits those who can run full nodes, issue transactions, execute smart contracts and read transaction history to approved computers and users. This feature therefore increases the integrity of the system, as well as guarding against hackers, and strengthens the system beyond its robust foundation of public-key cryptography.

Taking into account these concerns, a more practical solution than expecting patients themselves to take control of their lifelong health record increasingly seen in the field, is an independent company-managed electronic health record database. These typically employ Blockchain to secure patient data and to empower patients in a way that has not previously been possible.

The concept of Blockchain-based medical record management has been considered and implemented on a small scale by a number of companies^7,31,32^; however, only very few healthcare systems have begun to incorporate the technology into their nationwide infrastructure. Of those, Estonia is at the forefront, securing more than one million citizens’ records in a ledger in collaboration with Guardtime. The system has proven that interoperability is an achievable goal, and demonstrated that the ability to analyse data has helped the government to become aware of and more easily track health epidemics^33^.

Additional benefits of using Blockchain for health records include the ability to analyse the information with artificial intelligence. This will be more easily able to determine population trends, which can be used to achieve population level health. However, it will require careful integration, to allow sufficient integration without compromising privacy of patient data or security against hackers.

Further, data gathered from mobile applications, wearable sensors and other recent forms of technology could also contribute important information to the system, allowing physicians to create specialised treatment plans based on more frequent data. This is increasingly possible in an environment where continuous and detailed data is already being collated by the Internet of Things. It is also thought that such continuous health data would engage a patient more in their health care, improving compliance and long-term outcomes. Open source software means that different health IT systems could integrate the use of Blockchain as they wish, making this a versatile opportunity. The use of wearable technology and the incorporation of the Internet of Things into AI-based data analysis would bring forth additional benefits with a larger index of accurate data points.

Some administrative matters must be considered when implementing a Blockchain. Removing duplicates when consolidating legacy systems is costly and time-consuming. Once in place, it is vital that users of the system input good quality information; otherwise, the trustworthiness of the system arising from Blockchain’s immutability and decentralisation give way to the lack of accurate information, creating a critical point of failure. Nevertheless, the costs associated with educating users on how to make the most use out of the system would lead to returns in health outcomes. In the primary stages, usefulness will still depend on the end user experience, and so the requirement of hiding the complexities of Blockchain behind a sufficiently user-friendly interface becomes paramount to ensuring successful uptake. These primary stages will establish the most effective systems.

**Fig. 3:**
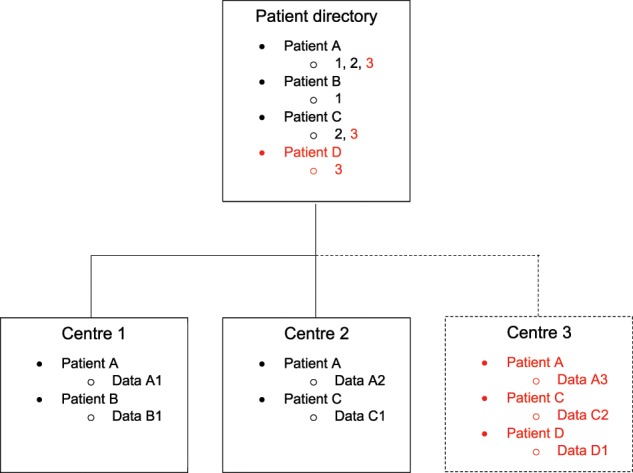
Connecting independent healthcare providers. Each box represents a Blockchain ledger. The Patient Directory lists all pseudonymised patients about whom data is stored across all providers’ Blockchain ledgers. Associated with each patient is a pointer to all centres (represented by number) where that individual has had a medical interaction. Individual Centres store their own ledgers, containing more detailed metadata (represented by code, e.g. Data A1, which does not imply storage of sensitive medical information) about interactions with patients, including associated cloud-based data storage locations and access permission information. When a Centre joins the system (e.g. Centre 3), basic pseudonymised information about its patients is relayed to the Patient Directory, allowing other Centres to access that information, subject to any associated smart contract-based permissions.

As various healthcare providers and companies update their record management systems on different timescales, it is necessary to consider how multiple ledgers might interact with one another. We outline below one potential framework to demonstrate the integration of several Blockchain ledgers managed by independent healthcare providers (Fig. 3). Another architecture would involve a system of records managed by independent companies on behalf of patients, with healthcare providers given data access but not the privilege of management. In either case, as individual providers introduce their own ledger systems using Blockchain’s API, they could connect to a wider network of Blockchain-based providers, allowing patients to visit different hospitals, or switch to a different healthcare data management company. This would allow doctors more easy access to a comprehensive set of data, with the patient’s explicit consent.

## Conclusion

A Blockchain allows data across multiple independent systems to be accessed simultaneously and immediately by those with sufficient permissions. This interfacing of different systems saves medical and financial sacrifices and reduces administrative delays. The use of smart contracts allows patients’ consent preferences to be executed immediately, further reducing administrative costs. An off Blockchain data lake is scalable and can store a variety of data types, making it versatile and suitable enough for the developing forms of data brought about by the Internet of Things in the healthcare field. Furthermore, it supports high-throughput data analysis as well as machine learning strategies to be applied, while being encrypted and digitally signed to ensure data privacy and authenticity of access. Interoperability achieved in this manner will allow greater collaboration between patients, doctors and researchers, leading to specific and personalised care pathways.

Its weaknesses must however be taken into account during development: Blockchain involves concepts unfamiliar to the vast majority of the population, including cryptographic signatures and key management. Costs are involved in concealing these and assimilating data from various legacy systems while maintaining adherence to various regulatory restrictions.

Nevertheless, Blockchain represents an innovative vehicle to manage medical records, ensuring interoperability but without compromising security. It also protects patient privacy, allowing patients to choose who can view their data. Investments into this technology would be outweighed by returns as the interfacing of systems leads to increased collaboration between patients and healthcare providers, and improved healthcare outcomes.

### Exclusive license

The authors grant to the Publishers and its licensees in perpetuity, in all forms, formats and media (whether known now or created in the future), to (i) publish, reproduce, distribute, display and store the Contribution, (ii) translate the Contribution into other languages, create adaptations, reprints, including within collections and create summaries, extracts and/or abstracts of the Contribution and convert or allow conversion into any format, including without limitation audio, (iii) create any other derivative work(s) based in whole or part on the on the Contribution, (iv) to exploit all subsidiary rights to exploit all subsidiary rights that currently exist or as may exist in the future in the Contribution, (v) the inclusion of electronic links from the Contribution to third-party material wherever it may be located and (vi) licence any third party to do any or all of the above.

### Transparency declaration

The authors declare that the manuscript is an honest, accurate, and transparent account of the study being reported; that no important aspects of the study have been omitted; and that any discrepancies from the study as planned (and, if relevant, registered) have been explained.

## Data Availability

This manuscript summarised information from publicly available literature. Any questions on source data can be forwarded to the corresponding author.
